# From Engineered Stone Slab to Silicosis: A Synthesis of Exposure Science and Medical Evidence

**DOI:** 10.3390/ijerph21060683

**Published:** 2024-05-27

**Authors:** Chandnee Ramkissoon, Sharyn Gaskin, Yong Song, Dino Pisaniello, Graeme R. Zosky

**Affiliations:** 1 Adelaide Exposure Science and Health, School of Public Health, University of Adelaide, Adelaide, SA 5064, Australia; sharyn.gaskin@adelaide.edu.au (S.G.); dino.pisaniello@adelaide.edu.au (D.P.); 2 Menzies Institute for Medical Research, College of Health and Medicine, University of Tasmania, Hobart, TAS 7000, Australia; yong.song@utas.edu.au (Y.S.); graeme.zosky@utas.edu.au (G.R.Z.)

**Keywords:** engineered stone, crystalline silica, ban/prohibition, occupational exposure, host characteristics, silicosis, narrative review

## Abstract

Engineered stone (ES) is a popular building product, due to its architectural versatility and generally lower cost. However, the fabrication of organic resin-based ES kitchen benchtops from slabs has been associated with alarming rates of silicosis among workers. In 2024, fifteen years after the first reported ES-related cases in the world, Australia became the first country to ban the use and importation of ES. A range of interacting factors are relevant for ES-associated silicosis, including ES material composition, characteristics of dust exposure and lung cell-particle response. In turn, these are influenced by consumer demand, work practices, particle size and chemistry, dust control measures, industry regulation and worker-related characteristics. This literature review provides an evidence synthesis using a narrative approach, with the themes of product, exposure and host. Exposure pathways and pathogenesis are explored. Apart from crystalline silica content, consideration is given to non-siliceous ES components such as resins and metals that may modify chemical interactions and disease risk. Preventive effort can be aligned with each theme and associated evidence.

## 1. Introduction

The rapid rise in the number of identified cases of engineered stone (ES)-associated silicosis is a global occupational health disaster [1]. Silicosis is one of the oldest known occupational diseases, with detailed case series published more than a century ago and with well-established and effective occupational hygiene interventions [1]. Thus, the observation that an entirely preventable lung disease has re-emerged in modern workforces is alarming. In addition, the pathogenesis of ES-associated silicosis is such that the disease is often more severe than historical cases of silicosis, with a shorter duration of exposure and a high incidence of progressive massive fibrosis [2]. These health concerns, combined with a high prevalence of the disease among ES workers (up to 28% in one cohort) [3], has led some jurisdictions in Australia to ban ES [4]; this is an extreme response, but one that is at the highest level of the hierarchy of controls for occupational health and safety.

There has been considerable debate as to the main drivers of disease severity and prevalence amongst workers fabricating ES benchtops. Early studies considered fabrication practices (e.g., wet versus dry cutting) [5], dust exposure levels [5] and the high level of crystalline silica [6] in many of the slabs (unfinished products) on the market. However, more recent studies have shown that the situation is more nuanced than this with evidence that fabrication of ES finished products leads to the emission of vapours (e.g., volatile organic compounds (VOCs) [7] and airborne particulate matter other than silica [8] that pose a threat to respiratory health. In addition, the over-representation of immigrant workers amongst silicosis cases [3] suggests that there are non-material-related factors contributing to disease risk.

**Figure 1:**
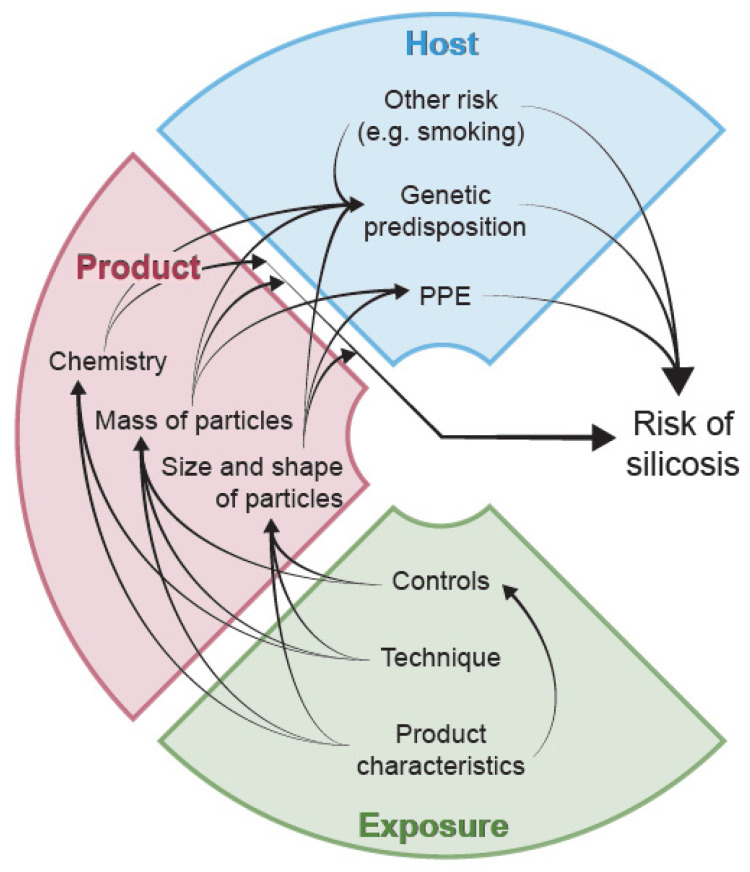
The risk of developing silicosis as a result of inhalation of emissions from the fabrication of engineered stone is a complex result of the interaction between the fabrication process (including the controls in place), the physico-chemical properties of the product and factors related to the individual (host) who is exposed.

Thus, the risk of developing ES-associated silicosis is complex, but appears to be a combination of the physico-chemical nature of the slab material, the emissions generated and inhaled during fabrication and host-related factors (Figure 1). The aim of this narrative review is to bring together evidence across disciplines from material and exposure science to laboratory toxicology and clinical studies in patients. The objectives are to characterise pathogenesis, identify gaps in knowledge, research needs and opportunities to reduce the burden of this preventable disease in industry and the community.

## 2. The Unfinished Product

### The Constituents

Engineered stone (ES) is an artificial stone material produced by agglomerating a high percentage of small particles with a small percentage of binder under “vacuum-vibro” compaction to make large uniform slabs [9]. In the 2000s, ES was produced by combining finely crushed material of >90% *w*/*w* crystalline silica with polymeric resins, pigments and additives to produce slabs of various colours and patterns for benchtop and cabinet making [9,10]. The forms of crystalline silica identified in ES are quartz (most common) as well as cristobalite [6], and the types of resins used for ES manufacture include polyester-styrene, acrylic, epoxy, methacrylate polyethylene [11] and polyurethane resins [12].

Not all products previously labelled as ES contain organic binding resin. Some products are essentially ceramic—composed entirely of inorganic raw materials (clays, feldspars and silica) pressed together at high temperatures (typically 1200–1400 °C in a sintering process) and high pressure to form ultracompact surfaces, mimicking the metamorphic formation of natural stones such as granite and marble. Commercially popular examples include Dekton^®^ and Neolith^®^, which are marketed as sintered stone. Some products are alternatively classified as porcelain, if they are only heated to high temperatures to achieve partial vitrification.

ES benchtops owe their popularity to their durability, aesthetic, ease of installation, resistance to chemicals and heat, and cost-effectiveness, particularly in comparison to natural stones (NS) such as marble and granite. This explains their rapid growth in the market. In Australia, ES was used for 60% of the >200,000 residential kitchen and >400,000 bathroom benchtops installed during the financial year 2021 alone [13]. 

The uncontrolled processing of high crystalline silica-containing ES materials generates high levels of respirable crystalline silica (RCS) and possibly a whole suite of other respiratory hazards [6,8,10]. Hence it is perhaps not surprising, albeit still tragic, that the growth in the ES industry has seen a concurrent rise in occupational lung disease among workers in the stone benchtop industry. In 2009, cases of silicosis outbreaks among ES workers started to be reported in countries like Israel [14] and Spain [15]. In Australia, the first case of ES-associated silicosis was reported in 2015; since then, >570 cases have emerged [16]. In a recent Australian study looking at disease prevalence among a large cohort of stone benchtop industry workers, a staggering 28% of screened workers were diagnosed with silicosis [3]. 

These health concerns have driven stone manufacturers around the world to re-think their stone formulation, without compromising durability, strength and aesthetics. This has resulted in the emergence of *reduced-silica ES* on the market in recent years. These reduced-Si ES products typically contain a mixture of <10–50% crystalline silica, up to 90% recycled materials (often recycled glass), assorted minerals, polymeric resins and typically <5% additives and pigments [17,18]. 

More recently, in line with a full prohibition on the use of silica-containing ES in Australia, stone manufacturers are also developing *zero-silica*/*crystalline silica-free ES* products. Major constituents (>70% reported in safety data sheets) of these artificial zero-silica stone benchtop products are recycled glass [19,20], aluminium trihydrate [21] and to a smaller extent, organic fillers such as resins, pigments and trace metals. 

## 3. Fabrication Processes

Once large stone slabs are manufactured, they are transported to local stone workshops where they are further processed into bespoke designs to meet customer demands [22]. Typically, ES processing involves the use of power tools to cut, drill, grind and shape the stone slab into benchtops. These are then polished and laminated to achieve the final product [23]. Some of these tasks are carried out semi- or wholly-automated and in enclosed conditions, e.g., cutting using waterjet cutting or computer numerical control (CNC) machines, while tasks like polishing are often carried out manually using pneumatic hand-held power tools. However, smaller workshops can often have more tasks carried out manually [23], likely due to the lack of infrastructure required for automation. Most of these tools are operated under wet conditions, using recycled water systems ranging from commercial filtration set-ups to in-house settling tanks to feed on to semi-automated and automated tools. Hand-held tools such as pneumatic grinders and polishers are typically centrally water-fed through hose attachments. After benchtops are cut and polished in stonemasonry workshops, they are transported to customers’ premises for onsite installation, where finishing touches such as smoothing of edges or holes are performed using hand-held tools such as angle grinders—which is often a dry process [24].

Benchtop making can be a dynamic process, involving a variety of tasks including cutting, shaping, set-up time and moving slabs during a work shift [25]. As such, there may be multiple workers doing different tasks contributing to the dust atmosphere in the workplace. This represents a challenge for accurate exposure assessments to determine the most at-risk tasks in the workplace and such situations may concomitantly place place at risk workers not actively generating dust. It is also noteworthy that while uncontrolled (i.e., dry) processing of ES is not permitted by many countries, including Australia, workers still resort to brief dry methods for specific operations, for example during benchtop installations, as described earlier [5,23,25]. Brief dry processing ES can cause extremely high levels of exposures to RCS [26].

### 3.1. What Is Emitted during Fabrication?

The emissions generated through processing ES have been well characterised in terms of the size, mass, and physico-chemical characteristics of associated respirable dust (RD) and respirable crystalline silica (RCS), as well as other airborne hazards. These emissions are often a function of the fabrication task, tools used and control measures applied during the task. 

#### 3.1.1. Respirable Dust (RD) and Respirable Crystalline Silica (RCS) 

Comparing RD and RCS levels generated through processing several types of stones (resin-based and sintered ES, and NS), Hall et al. (2022) [11] showed that the levels of airborne dust in different size fractions (inhalable, thoracic and respirable fractions) are comparable among the stones, but the levels of RCS were reflective of their bulk crystalline silica composition. This means that processing traditional high-Si ES (>90%) generated high levels of RCS (up to 80% by weight of RD), and accordingly, processing reduced-Si ES products generated lower levels of RCS [8]. This also applied to NS, for example, with processing sandstone (62% silica by weight) generating up to 55% by weight RCS [11], although other studies [27,28] observed that granite (NS) generated more respirable dust per unit volume of material compared to ES.

Both quartz and cristobalite make up the total RCS content of ES emissions. Quartz tends to be the predominant silica form of ES emissions, but certain samples contain more cristobalite than quartz [6,8,11,23,27,28]. Negligible levels of tridymite have been observed in ES emissions [11]. This confirms that air (dust) monitoring for RCS workplace exposure assessments should include all forms of crystalline silica. In comparison, NS such as granite only generate quartz when fractured [27]. 

#### 3.1.2. Particle Size, Mass and Morphology

ES processing generally generates small particles, with many studies reporting that >90% of the airborne particle mass is in the respirable fraction (4 µm mass median aerodynamic diameter) [6,29] with some mass even in the nanometre range (<100 nm in aerodynamic diameter) [28]. Fabrication tasks influence the size of the emitted particles. During cutting, a higher amount of airborne dust is produced compared to when stones are polished; during polishing, the size distribution of the particles is smaller and narrower [11]. No clear difference in normalised particle size distributions have been observed between ES and NS particles emitted during cutting or polishing [11,27].

ES particles generated by either wet or dry processing have conchoidal fractures, sharp edges and irregular shapes, characteristic of fractured pure quartz crystals. ES particles also show aggregation, whereby small particles stick to the surface of larger ones, potentially by static charges, as shown on images generated using scanning electron microscopy by Pavan et al. (2016) [29] and Ramkissoon et al. (2022) [6]. In comparison, it has been observed that NS particles generated in a similar way as ES exhibited less fractures at the surface, or sharp edges, and are more ‘layered’ structures [6].

#### 3.1.3. Volatile Organic Compounds (VOCs)

Many ES, either ‘traditional’ high-Si ES or new-generation reduced-Si ES, contain on average 5–15% by weight resin [8,30]. Processing these resin-based ES generates a suite of VOCs, many of which have been independently linked to adverse lung health outcomes in the literature. One of the most common VOC identified from ES processing is styrene [7,10], which has important health implications as occupational exposure to styrene has been directly linked to pulmonary toxicity [31]. Other commonly found VOCs are toluene, benzene, polychlorinated biphenyls (PCBs), polyaromatic hydrocarbons (PAHs) and respiratory sensitisers such as phthalic anhydride [8,10,11]. 

Most studies reporting ES-associated VOCs have reported semi-quantitative data under laboratory test conditions, which therefore cannot be directly related to worker exposure to VOCs during fabrication tasks. Reed et al. (2019) [32] were among the few ones to report onreal-world full-shift occupational exposure monitoring of VOCs during ES manufacture. Similar to laboratory studies, they reported a variety of VOCs, including styrene and other “aliphatic and aromatic hydrocarbons, ketones and alcohols emitted from thinners/solvents, glues/adhesives, resins and other industrial chemicals used on the premise”, that ES workers may be exposed to during stone benchtop manufacture [32]. The processing of ES often entails workers complaining of smells, attributable to the breakdown of resin. However, Reed et al. (2019) [32] did not report exposures above the workplace exposure standards.

#### 3.1.4. Metal Composition

Among the inorganic constituents of ES are metal elements, originating most likely from pigments that are added during ES manufacture [33]. Studies have reported between 11 and 17 metals in variable amounts in ES dust samples [8,10,29]. Among the most common and abundant metals (>1 g/kg) are Al, Na, Fe, Ca, and Ti. Tungsten (W) has also been reported to be a major component of ES, although it cannot be verified whether this is intrinsic to ES or an artefact as a result of contamination from the blade used for cutting (steel and carbide bits) [10]. Other elements such as Co, Cu, Ni, Zn and Ba have been identified in concentrations ranging from 3 to 312 mg/kg, while more toxic metals such as As, Cd and Pb are typically <0.2 mg/kg (except Pb which ranged from 0.53–409 mg/kg) [10]. 

It is noteworthy that natural stones contain much higher levels of metals, compared to ES. We showed that natural stones such as granite and marble contain metals between 29 and 37% by weight of their total composition. White granite and marble contain predominantly alkaline metals such as Ca, Mg and Al while black granite shows more variation in its metallic elements, including transition elements such as Ti and Fe [6].

### 3.2. How Do Their Constituents Impact on What Is Emitted?

It is generally agreed that the bulk composition of ES is representative of the emissions generated. For example, processing high-Si ES generates high levels of RCS dust, which in turn is proportionately lower when reduced-Si ES products are processed [8,27]. There is also evidence of other ES bulk constituents being made airborne during active processing, including transition metals like Ti and Co [8,34] and VOCs as a result of resin decomposing when ES is cut [7,11]. 

Many of the new-generation reduced-Si ES contain recycled materials such as glass (amorphous silica) and ceramic wastes, bound together with polymeric resins. Some manufacturers of ES have also reported feldspars in their production. Feldspars are minerals containing aluminium and varying amounts of potassium, sodium, and calcium [35,36]. While there is limited evidence of comparable exposure outcomes for the different types of ES, it seems perhaps reasonable to expect their bulk constituency to be reflected, to some extent, in their emissions. 

Evidence also suggests that processing resin-based ES at high temperatures can lead to more variable hazard emissions and potentially more diverse health effects compared to sintered ES, which have already been subjected to high temperatures during manufacture. An example is the VOCs produced when processing resin-based ES [8,11]. In the case of sintered ES, which is harder than resin-based ES, there is uncertainty as to whether the additional mechanical energy and different abrasive action required in processing sintered stone results in different particle characteristics. Both Carrieri et al. (2020) [28] and Thompson and Qi (2022) [27] reported that reduced-Si sintered ES containing predominantly aluminosilicates (clays, feldspars) and recycled glass, respectively, and generated similarly fine particles (<1 µm in aerodynamic diameter) as ‘traditional’ high-Si ES during cutting. 

This is an important future research direction as emerging ES benchtop products containing inorganic waste, recycled glass and amorphous forms of silica, rather than crystalline silica, may still pose a risk to worker health. More research is needed to understand the composition of dusts generated by processing new-generation reduced and zero-silica resin-based, and sintered ES products.

### 3.3. Workplace Exposure Controls

The extent of health risks from crystalline silica are associated with airborne silica dust exposure, not simply the crystalline silica content of the bulk material. Silica dust exposure is in turn determined using several additional factors such as manufacturing processes and dust control measures in place that influence the overall RCS dust generation. For example, in the ES context, silica exposure intensities have been defined as the proportion of time using ES and dry processing (without water suppression) [5]. 

This section provides an outline of what is known about the relative efficacies of exposure controls according to conventional occupational hygiene practice. The Australian situation provides context.

Beyond what is now being mandated in Australia by the model occupational health and safety Regulations, a combination of control measures is often advised for controlling RCS exposures to below the current Australian 8 h Workplace Exposure Standard (WES) of 0.05 mg/m^3^ to prevent disease. To date, regulatory action in Australia typically entailed the prohibition of uncontrolled dry abrasive processing, the use of integrated tool vacuum systems, wet methods, and fit-tested personal respiratory protection. 

Controlling exposures to hazards in the workplace is vital to protecting workers. The so-called *hierarchy of control measures*, as described in Regulation 36 of the Model Work Health and Safety (WHS) legislation, can be applied to this occupational health problem. The hierarchy of controls is a way of determining which actions will best control exposures through five levels of actions to reduce or remove hazards. The preferred order of action based on general effectiveness is: Elimination, Substitution, Engineering controls, Administrative controls and Personal protective equipment (PPE) [37]. Relevant available literature evaluating control measures in engineered stone and related industry settings is presented in Table 1.

The world-first ban on the use, supply and manufacture of ES in Australia represents the application of *Elimination* as the highest form of control in the hierarchy (removing the hazard at the source) to supplement existing controls. Evidence is already seen of industry pivoting in response to the ban. Next generation ‘low/reduced-’ to ‘zero-silica/silica-free’ products are becoming available on the market (see section The Unfinished Product—The constituents), and porcelain and ceramic products are exempt from the ban. In effect, this represents *Substitution* in the hierarchy of controls, as benchtop products will still be available. Effective substitution involves using a safer alternative to the source of the hazard. However, when considering a substitute, it is important to compare the potential new risks of the substitute to the original. New research into the emissions from these emerging products is needed to understand the relative risks compared with their high-silica ES predecessors as well as ‘traditional’ natural stone products such as granite and marble.

*Engineering* control measures reduce or prevent hazards from coming into contact with workers and can include modifying equipment or the workspace, using protective barriers, ventilation, enclosing/isolating processes and more. Examples commonly used in the ES industry have included the on-tool water suppression of dust (wet processing) and/or dust extraction devices such as on-tool dust extraction or local exhaust ventilation (LEV). Water jet cutting via the use of CNC machines is applicable for the factory setting, but not for on-site installation. In fact, automation (e.g., CNC machines) have been shown to result in lower exposures than hand tools due to the operator position being further from the cutting edge. Local Exhaust Ventilation (LEV), particularly on-tool LEV, has previously been shown to be effective in respirable dust control in concrete grinding and polishing [38,39,40]. 

A combination of wet process methods and LEV can suppress dust more effectively, by a factor of 10 or more, compared to if a single control measure was applied [41,42]. For example, Cooper et al. (2015) [41] reported short-term (30 min) RCS levels of 44 mg/m^3^ for dry activities, which reduced to 4.9 mg/m^3^ through the adoption of wet methods and further reduced to 0.6 mg/m^3^ when the latter measure was combined with LEV. This is because workers using predominantly wet methods may still carry out brief dry operations, for example, during finishing processes such as smoothing the edges or holes. Dry work is not uncommon during in-home installation of benchtops, where water suppression methods may not be available, and finishing tasks end up being carried out manually (i.e., dry finishing). Glass et al. (2022) [5] observed that, even though there has been a 5-fold reduction in the proportion of time that ES workers spend on dry operations from their earliest job to the most recent, 16% of them still reported dry-processing at least 50% of the time. Recently, Weller et al. (2024) [23] also found that despite all studied worksites using wet methods for ES processing, a substantial number of workers were likely exposed to RCS above the WES if not wearing respiratory protection. Wet dust suppression methods *in combination* with on-tool LEV may therefore be required to prevent overexposure to RCS while working with ES [26,43,44]. 

Qi and Echt (2021) [45] reported significant improvement with the addition of sheet-wetting engineering control when hand grinding ES. They noted that neither the centre feed nor the water spray method provided an effective wetting of the active grinding spot on the stone countertop. This resulted in partially dry grinding during the operation. Similarly, our previous work [46] simulating tasks with pneumatic grinders and polishers also found that water jets on the grinder were not consistently and uniformly contacting the cutting surface, and this resulted in variable dust suppression outcomes. Salamon et al. (2021) [24], in a study of four facilities in Italy, found LEV to be effective in wet and dry finishing and stated there was a remarkable reduction in RCS exposures.

*Administrative* controls establish work practices that reduce the duration, frequency, or intensity of exposure to hazards, and should only be used to provide additional protection after implementing substitution and engineering controls. Administrative controls rely on worker behaviour and include work practice policies, training, and good housekeeping, among others. There has been limited evaluation of interventions to improve the knowledge and practice for silicosis prevention amongst high-risk workers [47,48]. The knowledge, attitudes and behaviour of workers in workplaces is further discussed in section *The Host*. Education and training interventions must consider workers’ demographic factors that can influence their effectiveness, with high proportions of culturally and linguistically diverse and vulnerable migrant workers represented in this industry in Australia [49] and likely elsewhere. 

Like administrative controls, *PPE* requires consistent and ongoing effort by workers and their supervisors to effectively control exposure. Limited data are available on PPE (specifically respiratory protection) performance in relation to ES dust exposure control. Respiratory protective equipment can be an unreliable control measure and failure to conduct respirator fit testing on workers has been a frequent problem in this industry [23,50]. Recently, Weller et al. (2024) [23] reported that Class P2 or N95 respirators providing an assigned protection factor (APF) of 10 (in accordance with Standards Australia/Standards New Zealand AS/NZS 1715-2009) provided adequate protection for workers who were clean shaven and fit tested, when used in combination with wet dust suppression methods. The impact of facial hair on respirator protection performance has also been highlighted as an issue in compliance programs [51]. Weller et al. (2024) [23] suggested that powered air-purifying respirators (PAPR-P3) provide a higher APF of 50 and may overcome the issue of workers with facial hair while also being more comfortable, especially in summer. One challenge of respiratory protection use is that workers doing hand polishing or grinding wearing PAPRs can have their visibility obscured by water splashing onto the visor, which may impact use compliance.

Overall, there currently does not appear to be consensus on ‘best practice’ for effective wet methods for dust control or LEV to allow for the capture of dust at the source. Regardless of the silica content of the bulk ES product being processed, it will continue to be vital to adopt multiple control measures, used in combination (e.g., wet-cutting and appropriate fit-tested respiratory protection) when processing ES to reduce exposure.

**Table 1:** Literature examining the use of engineering controls such as wet suppression and local exhaust ventilation in industries related to engineered stone. Presented in chronological order, RCS values represent Geometric Mean.

Reference	Industry, Material/s and Tasks	Control Measures Studied	Major Outcomes
Croteau et al., 2002. [38]	Construction. Dry tuck-point grinding, concrete surface grinding, angle grinder, paver block and brick cutting (masonry saw), concrete block cutting (hand-held saw). Continuous 15 min each task (controlled simulation). Personal and area sampling.	LEV: (on-tool shrouds) ventilation rates 0, 30, 75 cfm	LEV reduced personal exposure levels to RD (and RCS) by 85–99%: 22.17 mg/m^3^ to 3.01 mg/m^3^ RD, and 3.04 to 0.47 mg/m^3^ RCS during tuck-point grinding; 165.34 to 8.00 mg/m^3^ RD, and 29.16 to 1.70 mg/m^3^ RCS during surface grinding; 89.95 to 4.31 mg/m^3^ RD, and 22.25 to 0.95 mg/m^3^ RCS during paver cutting; 26.69 to 3.67 mg/m^3^ RD, and 4.24 to 0.60 mg/m^3^ RCS during brick cutting. Protected workers during short duration work tasks (and bystanders). Reduced clean up.
Healy et al., 2014. [40]	Stonemasonry/restoration. Grinding sandstone; 5-inch angle grinder. Tool (cup grinder) ~4000 RPM 15 min each task (controlled simulation) (10 min no shroud). Personal sampling.	LEV: 4 x on-tool shrouds (FLEX, Dust Muzzle, Dustie, Hilti)	LEV reduced RD personal exposure when grinding by 92% (7.1 to 0.5 mg/m^3^) and RCS by 99% (4.2 to 0.03 mg/m^3^) (all data).
Cooper et al., 2015. [41]	Engineered stone (85% quartz), slab 1.4 m × 0.8 m × 19 mm. Handheld worm-drive circular saw. Simulated in 24 m^3^ tent (3.1 m × 3.1 × 2.1 (2.7 vaulted roof)). Performed 4 × 30 min trials, 6 mm deep 3 mm wide cuts (27 cuts total). Order of trials randomised within each replicate block. Total of 3–5 trials per day with periodic rinsing of the area. Personal sampling. One field blank per day.	Wet blade; wet blade + water curtain spray; wet blade + LEV	Mean quartz content of respirable dust was 58%. RD and RCS: Dry cutting 69.60 mg/m^3^ (RD) and 44.37 mg/m^3^ (RCS). Statistical difference (by a factor of 10) between RD exposure using wet blade only (4.934 mg/m^3^) and wet blade + LEV (0.225 mg/m^3^), but not between wet blade only (4.934 mg/m^3^) and wet blade + curtain 3.813 mg/m^3^). Similarly for RCS, 4.934 mg/m^3^ for wet blade only, 3.813 mg/m^3^ wet blade + curtain and 0.604 mg/m^3^ wet blade + LEV.
Johnson et al., 2017. [42]	Engineered stone fabrication. Cutting, grinding, polishing, drilling. Grinder ~10,000 RPM Polisher ~4000 RPM Trial 20 min (controlled experiments). Personal sampling.	Wet (sheet-flow); On-tool LEV; Wet + LEV	Sheet-flow wetting + on-tool LEV during cup wheel grinding effectively reduced RCS by 50%; 1.128 mg/m^3^ with LEV, 2.115 mg/m^3^ without LEV. Water-spray-wetting on grinding cup LESS effective when combined with LEV! That is, 2.988 mg/m^3^ with LEV, 0.434 mg/m^3^ without LEV.
Enis, C.B., 2017. [52]	Engineered stone (Caesarstone; <93% quartz and <50% cristobalite), 2 cm thick slab. Simulated/controlled grinding using hand-held 10cm (electric) grinder with diamond cup wheel (~9000 RPM). 20 min controlled trials (*n* = 3–4); 45-degree edge grinding. Personal sampling. Total of 5 field blanks each day.	Combination of controls: Low flow LEV (one vac), High flow LEV (two vac), Sheet flow wet no LEV, Sheet flow wet with low flow LEV, and Sheet flow wet with high flow LEV.	RD: Dry grinding, High flow LEV (6.11 mg/m^3^) more effective than Low flow LEV (13.85 mg/m^3^) Sheet flow wet grinding; with High flow LEV (0.90 mg/m^3^) and Low flow (1.39 mg/m^3^) more effective than Sheet flow wet no LEV (3.83 mg/m^3^). Sheet flow wet High flow LEV not significanly different to Sheet flow wet Low flow LEV.
Reeves, T., 2018. [53]	Engineered stone and granite. Wet polishing (in a workplace) using sheet-flow wetting. Sample length 40–150 min only. Personal sampling.	Ventilation: Retractable paint booth (on/off)	19–81% (mean 57%) of respirable dust was RCS. No significant difference between on/off operation, but qualitatively lower when booth operating (RCS 73.62 µg/m^3^ booth off, 34.72 µg/m^3^ booth on).
Saidi et al., 2020 [54]	“Granite” polishing (dry). Simulated test bench and LEV. NaCl particles used to “simulate granite particle”. Area/static sampling.	LEV: push-pull system, dust shroud, tool integrated with suction slots	No specific particle concentrations provided, results presented only as %efficiency. All LEV systems effective, up to 95% reduction in NaCl. Confirmed that suction flowrate and speed of rotating discs influenced LEV performance.
Qi and Echt 2021. [45]	Workplace exposure assessment at three facilities processing (including grinding task) engineered stone. Personal sampling and some area sampling.	Workplace wetting methods: Water spray from a nozzle on a grinder, Centre-feed built into grinder, Combination of water spray and sheet-wetting methods	Both water spray (190.4 µg/m^3^) and centre-feed (168.4 µg/m^3^) methods performed equally poorly at wetting the grinding spot and reducing worker’s RCS exposure during grinding, despite having very different water flowrates. Adding sheet-wetting significantly reduced RCS exposure (33.2 µg/m^3^).
Salamon et al., 2021 [24]	Workplace exposure assessment at facilities processing artificial/engineered stone. 51 personal RCS samples.	Workplace had existing engineering controls: Extraction wall booths; Aspirated benches.	RCS content in RD was 8–19%. General outcomes: Extraction booths good for minimising general ambient dust load but not necessarily reduced personal exposure. RD and RCS: Wet manual processing reduced RD exposure (0.352 mg/m^3^ dry, 0.082 mg/m^3^ wet) and RCS exposure (0.039 mg/m^3^ dry, 0.020 mg/m^3^ wet). Aspirated benches achieved high capture speed near finishing operations and significantly reduced RCS levels.
Weller et al., 2024 [23]	Workplace exposure assessment at facilities processing artificial/engineered stone. 123 personal RCS samples (34 static) across 27 workshops in Sydney.	All used wet methods of fabrication.	GM of pooled result for RD was 0.09 mg/m^3^ and 0.034 mg/m^3^ for RCS. The highest exposed workers with a GM RCS of 0.062 mg/m^3^ were those using pneumatic hand tools for cutting or grinding combined with polishing tasks. Workers operating semiautomated routers and edge polishers had the lowest GM RCS exposures of 0.022 mg/m^3^ and 0.018 mg/m^3^, respectively. The wearing of respiratory protection by workers remains necessary until further control measures are more widely adopted across the entire industry, e.g., reduction in the crystalline silica content of ES.

## 4. The Host

### 4.1. Worker Behaviours 

The risk of developing silicosis increases with increasing dust levels and exposure duration. Dust exposure control is the only way to *prevent* workers from developing silica-related diseases; as highlighted above, dust control measures range in their effectiveness according to the hierarchy of controls. However, the effectiveness of these methods is also dependent on how they are used and/or maintained by workers, which in turn is associated with the knowledge, attitudes and behaviour of workers in workplaces [55,56]. A range of studies in different countries such as Vietnam [57], India [58,59], Spain [60], and China [61] consistently demonstrated that educated workers who are given adequate information and training were likely to be aware of the harmful outcomes of occupational exposure to silica dust [56], and therefore more willing to comply with health standards and regulations [62,63]. Moreover, educating workers has a direct impact on their proper use of prevention practices such as the effective use of personal protective respiratory equipment, which may prevent worsening of pulmonary function due to reduced dust exposure [64]. Not surprisingly, workers who serve longer years showed increased positive attitudes toward personal health compared to those that had fewer service years [65], probably due to their increasing knowledge and experience in occupational health and safety. In addition, a recent study [49] has found that occupational hygiene surveys, by gathering information on work processes as well as worker demography (such as length of work in current job, age and first language spoken by each worker) in micro or small-sized enterprises, could be a powerful tool to identify key criteria for targeted preventive programs in ES fabrication.

### 4.2. Host Risk Factors

There are a range of host-specific factors that may contribute to the risk of developing ES-associated silicosis. Studies have shown that older age, lower body mass index (BMI) and smoking are potential risk factors for silicosis among stone benchtop industry workers [3]. While the average age of development of ES-associated silicosis is lower than historical forms of the disease, due to the shorter latency period, being an older worker in the industry is associated with increased the risk of having the disease. However, the effect of age as a determinant of disease risk needs to be treated with caution due to the confounding effect of the correlation between age and cumulative exposure. Similarly, it is unclear whether the association with low BMI and disease risk is causal or is simply a marker of the effect of silicosis itself on body condition. Further work is required to untangle these complex associations. 

The impact of smoking on silicosis development has been extensively studied. For example, a recent study that focussed specifically on workers exposed to ES dust found an association between smoking history and risk of disease [3], in line with work in pottery workers [66]. Another cross-sectional study of natural stone fabricators found that, while prolonged cigarette smoking was not significantly correlated with X-ray evidence of lung silicosis (OR 1.59, 95% CI 1.00 to 2.53) [67], there was evidence of additive effects of cigarette smoke and dust exposure on disease risk. Similar complexity was observed in a small cohort of workers processing ES, whereby smoking status was not associated with silicosis in ES workers, but was correlated with declines in lung function in those with disease [68]. 

This complexity may be due to the effect of smoking-related lung disease on spatial patterns of lung deposition of dusts. This is evident in an early study analysed the relationship between complete smoking/dust exposure histories and autopsies in 4456 gold miners between 1976 and 1981 [69]. The data showed that ever smoking was not associated with parenchymal silicosis, but was negatively associated with pleural silicosis [69]. 

While most of the aforementioned studies have focussed on disease risk, others have confirmed a link between smoke exposure and disease progression. The additive effects of silica dust exposure and cigarette smoking have been demonstrated in a range of lung diseases such as lung cancer, respiratory tuberculosis and pneumoconiosis [70,71], although the effects may differ in the specific diseases [71]. Two Chinese studies have suggested that cigarette smoking is responsible for an increased risk of mortality in individuals exposed to silica dust [70,72]. In line with these data, in vivo animal studies revealed that tobacco smoke exposure exacerbated crystalline silica-induced lung toxicity in rats [73].

Taken together, while acknowledging the complexities of disentangling the collinearity of the risk factors, the evidence suggests that smoking increases both the risk of developing silicosis following ES inhalation and also exacerbates disease progression and outcomes. 

### 4.3. Genetic Predisposition

Genetic polymorphisms in cytokines (e.g., *IL-1*, *IL-4*, *IL-6*, *IL-17*, *TNF-α*), inflammatory pathway genes (e.g., *NF-ĸB*) and inflammasomes (*Nalp3*, *caspase-1* and *IL-1β*) have been extensively studied and associated with susceptibility to silicosis and pneumoconiosis, which have been well summarised in two recent systematic reviews [74,75]. A Brazilian study found that rs1800469 polymorphisms in the *TGF-β1* gene and rs763110 polymorphisms in the *FASLG* gene were linked to the severity of silicosis [76]. In addition, given the essential roles of non-coding RNAs (ncRNAs) in the regulation of several cellular processes (e.g., transcription, post-transcriptional modifications, and signal transduction), single nucleotide polymorphisms (SNPs) in ncRNAs may also influence silicosis susceptibility. In this regard, a silicosis-related genome-wide association study (GWAS) was constructed to select candidate SNPs located on circular RNA [77]. Seven circular RNA -SNPs were identified, with variant A of rs17115143 (an allele located on *SNHG14*) being found to have a strong association with silicosis risk in the validation study (OR = 1.68, *p* = 0.032) [77]. SNPs in long non-coding RNA (lncRNA) were also screened using (GWAS) in relation to the risk of silicosis [78]. The variant A allele of rs1814521 in the lncRNA *ADGRG3* was associated with a reduced risk of silicosis (OR = 0.76, 95% CI = 0.62–0.94, *p* = 0.011), aligned with the observation that *ADGRG3* was down-regulated in silicosis cases [78]. However, these genetic association studies have been performed in a single population, and more studies are required to replicate these findings in different ethnic cohorts with a focus on stone benchtop industry workers.

## 5. Discussion and Conclusions

The aim of this review was to synthesise evidence across disciplines of exposure science and clinical and laboratory research to help navigate the burgeoning ES literature, and to identify the gaps in knowledge and opportunities to reduce the burden of ES-associated silicosis. It builds on the systematic review by Leso et al. (2019) [79] which called for further research about the hazardous nature of ES dusts, workplace exposure measurements and the effectiveness of protective equipment to best protect workers against overexposures to hazards and risk of disease in the ES industry. Our review of evidence highlighted the complexity of the topic through the interactions between the product(s) characteristics, the emissions generated and inhaled during fabrication as well as host-related factors, which all play a role in the risk of developing silica-related disease in this occupation. 

Current screening tests in Australia have identified that up to 1 in 4 ES workers has silicosis [3], and experts estimate that this number could be even higher than what is currently identified [4]. Most of these workers diagnosed with silicosis are younger than 35 years old [4], who go on to experience even more stress if unable to continue working and have to stop work permanently [80]. The crystalline silica content of ES has been a significant factor in disease pathogenesis among ES workers; reducing the crystalline silica content of ES is a first step towards better product stewardship that, when handled under stricter health and safety regulations and more stringent workplace controls, is likely to reduce exposure to silica dust and associated health risks. However, our recent in vitro study [8] also highlighted the contribution of other ES components, notably metals such as Co and Al, and possibly resin decomposition products (VOCs), to adverse lung cell responses. This highlights the variability and uniqueness of the hazards associated with ES fabrication [79,81]. Exposure to silica dust is traditionally regulated in Australia and elsewhere, but co-exposures to other hazards remain a point of concern in this industry. 

The world-first decision to ban the use and supply of ES containing >1% crystalline silica was based on the precautionary principle that dictates “action should be taken, even without clear evidence, when the potential adverse effects for man or the environment are serious” [4,82]. In doing so, Australia adopted the highest hierarchy of control—elimination—to reduce the risk of exposure to airborne crystalline silica, and prevent disease. However, in reality, these products are being rapidly *substituted* by alternative products, either (crystalline) silica-free resin-based ES or porcelain- or ceramic-based ES, among others. 

Future research therefore needs to focus on determining the health and safety risks associated with emerging stone benchtop alternatives. In particular, current gaps in knowledge relating to VOC exposures, particle emissions from sintered stone, airborne dust variability due to waste material ingredients, PPE performance and host factors such as silicosis-genetic associations amongst ethnic cohorts, need to be addressed. In terms of the medical aspect of this occupational health issue, more mechanistic and clinical data in ES workers are required to better understand the pathogenesis of disease, as all current medical studies are relying on traditional silicosis cases.

## Data Availability

All data are contained within this article.
